# PLS2 in Metabolomics

**DOI:** 10.3390/metabo9030051

**Published:** 2019-03-15

**Authors:** Matteo Stocchero, Emanuela Locci, Ernesto d’Aloja, Matteo Nioi, Eugenio Baraldi, Giuseppe Giordano

**Affiliations:** 1 Department of Women’s and Children’s Health, University of Padova, 35128 Padova (PD), Italy; eugenio.baraldi@unipd.it (E.B.); giuseppe.giordano@unipd.it (G.G.); 2 Fondazione Istituto di Ricerca Pediatrica Città della Speranza, 35129 Padova (PD), Italy; 3 Department of Medical Sciences and Public Health, University of Cagliari, 09042 Monserrato (CA), Italy; elocci@unica.it (E.L.); ernestodaloja@gmail.com (E.d.); nioimatteo@gmail.com (M.N.)

**Keywords:** projection to latent structures regression, PLS-DA, post-transformation of PLS2, orthogonally-constrained PLS2

## Abstract

Metabolomics is the systematic study of the small-molecule profiles of biological samples produced by specific cellular processes. The high-throughput technologies used in metabolomic investigations generate datasets where variables are strongly correlated and redundancy is present in the data. Discovering the hidden information is a challenge, and suitable approaches for data analysis must be employed. Projection to latent structures regression (PLS) has successfully solved a large number of problems, from multivariate calibration to classification, becoming a basic tool of metabolomics. PLS2 is the most used implementation of PLS. Despite its success, PLS2 showed some limitations when the so called ‘structured noise’ affects the data. Suitable methods have been recently introduced to patch up these limitations. In this study, a comprehensive and up-to-date presentation of PLS2 focused on metabolomics is provided. After a brief discussion of the mathematical framework of PLS2, the post-transformation procedure is introduced as a basic tool for model interpretation. Orthogonally-constrained PLS2 is presented as strategy to include constraints in the model according to the experimental design. Two experimental datasets are investigated to show how PLS2 and its improvements work in practice.

## 1. Introduction

The analytical platforms used for metabolomics investigations produce large and complex datasets. Since a single metabolite can produce more than one analytical signal and the perturbation of a specific pathway can generate variations in metabolite concentrations that are not independent, redundancy and multicollinearity are structurally present in the data. Moreover, the difficulty to collect a large number of samples and the possibility to simultaneously measure hundreds or thousands of features produce short and wide data matrices. As a result, discovering the hidden information is a challenge, and suitable approaches for data analysis must be employed.

Metabolomics has been developed within the field of analytical chemistry. For this reason chemometric tools have been originally applied and most of the literature about data processing and analysis is in the domain of chemometrics rather than in the one of statistics. The chemometric approach to data analysis is different from that of classical statistics. Specifically, classical models based on probability distributions (‘hard data modelling’) are substituted by algorithmic approaches where no assumptions about data distribution are made (‘soft modelling’ approach). Projection to latent structures regression (PLS) is one of these algorithmic approaches. It was introduced at the beginning of 1980s as a heuristic method for modelling relationships between two data matrices, one containing the predictors and the other the responses [1,2,3]. In a larger sense, PLS is nowadays used to indicate a family of techniques that perform regression using the latent space discovered submitting two data matrices, X and Y, to a suitable bilinear decomposition. NIPALS-PLS2 (indicated in the following as PLS2) was the first algorithm proposed to perform PLS regression. Other algorithms were later introduced. Statistically inspired modification of PLS (SIMPLS) [4] and undeflated PLS (UDPLS) [5] are alternative algorithms that, however, provide different models. The use of PLS2 in metabolomics has been promoted by its capability to handle several collinear and noisy variables, the possibility to interpret the models by simple graphical representations and its implementation in several commercial software. It has been successfully applied to solve a large number of problems, from multivariate calibration to classification problems. However, if, on one hand, PLS2 provides good predictive models, often outperforming other regression methods [6,7], on the other hand it shows some structural limitations when specific clusters or trends in the data, that are not related to the response, are present. In these situations, PLS2 is often driven towards wrong directions for projecting the data and produces inefficient data representations where too many latent variables are used. To overcome this unwanted behaviour, post-transformation procedures have been proposed [8,9]. Moreover, orthogonally constraints have been introduced in the maximization problem of PLS2 to take explicitly into account the structure of the experimental design [10].

If someone wants to solve a data analysis metabolomic problem by PLS2, it is not sufficient to simply know PLS2, but it becomes fundamental to know and use the state-of-the-art of this technique. Practitioners should take care in choosing the right number of latent variables to avoid too complex models. Again, they should check the linearity into the latent space between x- and y-scores, highlight the presence of irrelevant structured variation and decide how to take it into account in performing data modelling. The aim of the present study is to provide a comprehensive and up-to-date presentation of PLS2 focused on its application to metabolomics. It is not a review of all the available literature, but a discussion of the state of development of this technique.

The paper is structured as follows: Section 2 is dedicated to the PLS2 algorithm. Specifically, its main properties, the post-transformation procedure, the strategy used to introduce orthogonal constraints, and the case of non-linearity are discussed. In Section 3, the approaches used to interpret the model are illustrated. Two experimental datasets are investigated in Section 4, including the discussion of both multivariate calibration and classification problems. Concluding remarks are reported in Section 5.

### Notation

The common notation, where column vectors are indicated in lower case characters (e.g., a) and matrices in upper case characters (e.g., A), is employed. The transpose of the matrix A is $A^{t}$, the row vectors are denoted as the transpose of the columns vectors (e.g., $a^{t}$), the scalar product between the two vectors a and b is then indicated as $a^{t}b$ and the matrix product of the two matrices A and B as AB. The vector with all l elements equal to zero is denoted as ${\underline{0}}_{l}$, the matrix with all elements equal to zero as $\underline{\underline{0}}$ and the identity matrix of size n as $I_{n}$. The matrix obtained juxtaposing the two matrices A and B is [AB].

Let us assume that $X\in R^{N\times P}$ and $Y\in R^{N\times M}$ are two scaled and mean-centred data matrices (where the rows represent the observations and the columns the variables) representing the X-block of the predictors and the Y-block of the responses, respectively. Under this assumption, the scalar product of the linear combinations of the columns of X and Y can be interpreted as covariances (up to a constant factor).

## 2. The PLS2 Algorithm

The PLS2 algorithm was developed within the framework of the NIPALS algorithm by Wold and Martens at the beginning of 1980 [1,2,3]. In subsequent years, several algorithms have been introduced to implement PLS2 in the limit case of a single y-response [11,12], but the formulation in the case of multiple Y-response has maintained its original form until now. PLS2 is implemented in the NIPALS form in several commercial software for multivariate data analysis. However, a more in-depth investigation of the properties of PLS2 can be obtained introducing the different (but equivalent) algorithm presented in the following.

Given the matrix Y of the responses and the matrix X of the predictors, the aim of PLS2 is to calculate the matrix of the regression coefficients B that produces the linear regression model:

$$Y=XB+F_{A}$$

where $F_{A}$ is the matrix of residuals. Since no assumptions about the statistical distribution of the residuals are made, the term ‘model’ will not be used in the statistical sense of ‘model based on a set of probability distributions’, but to indicate a particular ‘matrix decomposition’.

**Figure 1:**
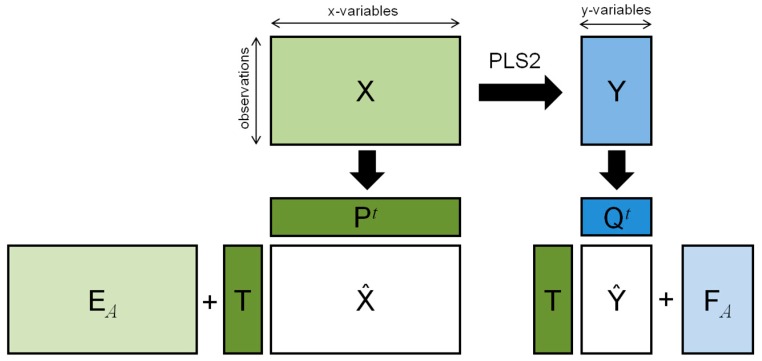
Matrix decomposition generated by PLS2.

PLS2 regression is performed decomposing the matrix of the predictors and that of the responses using a suitable orthogonal score matrix $T=\left[t_{1}\ldots t_{A}\right]$ as:

$$X=TP^{t}+E_{A}$$

and:

$$Y=TQ^{t}+F_{A}$$

under the constraint:

$$T=XW^{*}$$

where $W^{*}$ is a suitable matrix to be calculated, $P=X^{t}T{\left(T^{t}T\right)}^{-1}$ and $Q=Y^{t}T{\left(T^{t}T\right)}^{-1}$ the loading matrices of the X- and Y-block, respectively, and $E_{A}$ the matrix of the residuals of the X-block (Figure 1).

PLS2 linearly transforms the space of the predictors to obtain a new set of orthogonal variables (called latent variables whose values are the scores $t_{i}$), which can be used to model the response in the least-squares sense. In this way, the limits of ordinary least squares (OLS) regression due to redundancy and multicollinearity are overcome.

The matrix of the regression coefficients results:

$$B=W^{*}Q^{t}.$$

The core of PLS2 is the strategy used to calculate $W^{*}$. Following Di Ruscio [13] and Stocchero and Paris [8], $W^{*}$ can be calculated by the ‘eigenvalue’ PLS2 algorithm:

Given:

- two matrices X and Y;
- the integer number A of latent variables.
Let $E_{0}=X,\;F_{0}=Y$  For i=1,…,A    $E_{i-1}^{t}F_{i-1}F_{i-1}^{t}E_{i-1}w_{i}=\lambda_{i}w_{i}$            (1)    $t_{i}=E_{i-1}w_{i}$    $E_{i}={\hat{Q}}_{t_{i}}E_{i-1}\;\mathrm{X-deflation}\;\mathrm{step}$    $F_{i}={\hat{Q}}_{t_{i}}F_{i-1}\;\mathrm{Y-deflation}\;\mathrm{step}$EndFor

The matrix ${\overset{⌢}{Q}}_{t_{i}}=I_{N}-t_{i}{\left(t_{i}^{t}t_{i}\right)}^{-1}t_{i}^{t}$ is the orthogonal projection matrix that projects any matrix A onto the space orthogonal to the score vector $t_{i}$, whereas $E_{i}$ and $F_{i}$ are the residual matrices of the X- and Y-block at the step i, respectively. If the weight vectors $w_{i}$ used to project the residual matrix $E_{i}$ are arranged in the weight matrix $W=\left[w_{1}\ldots w_{A}\right]$, the matrix $W^{*}$ becomes:

$$W^{*}=W{\left(P^{t}W\right)}^{-1}$$

and the PLS2 model is obtained.

The explicit expression of the matrix B in terms of the weight matrix is:

(2) $$B=W{\left(W^{t}X^{t}XW\right)}^{-1}W^{t}X^{t}Y$$

If N>P and the columns of the design matrix X are mutually orthogonal, the matrix of the regression coefficients is equal to that of the OLS regression model, i.e., $B={\left(X^{t}X\right)}^{-1}X^{t}Y$, when one considers a number of latent variables equal to the number of variables of X. These conditions are satisfied for example in case of full factorial or fractional factorial designs.

In general, the matrix of the regression coefficients becomes:

$$B=VS^{-1}U^{t}Y$$

when the maximum number of latent variables is generated. Here, we have considered the singular value decomposition $X=USV^{t}$. Moreover, it is worth noting that the expression of the matrix B in terms of W for a model with A latent variables is the same obtained in case of constrained OLS where the constraint is $Q^{t}B=\underline{\underline{0}}$, being $Q:Q^{t}W=\underline{\underline{0}}$, rank(Q)=P−A and [QW] a non-singular matrix.

The ‘eigenvalue’ PLS2 algorithm is composed of two main ingredients: a well-defined eigenvalue problem to be solved (equation 1) and the iterative deflation algorithm, that corresponds to the reported algorithm when in 1 a general and non-trivial $w_{i}$ is used. The solution of the eigenvalue problem 1 solves the following maximization problem:

(3) $$w_{i},c_{i}\;s.t.\;\arg\max t_{i}^{t}u_{i}=\underset{\begin{array}{l} w_{i}^{t}w_{i}=1\\ c_{i}^{t}c_{i}=1 \end{array}}{\arg\max}\;w_{i}^{t}E_{i-1}^{t}F_{i-1}c_{i}$$

where $u_{i}$ is the score vectors of the Y-block obtained projecting the residual matrix $F_{i-1}$ along the unit vector $c_{i}$. The maximization problem can be read as: to find the weight vectors $w_{i}$ and $c_{i}$ that produce the score vectors $t_{i}$ and $u_{i}$ maximizing their covariance when used to project the residual matrices of the X- and Y-block, respectively. If $w_{i}$ is known, one has:

$$c_{i}\propto F_{i-1}^{t}E_{i-1}w_{i}$$

and:

$$u_{i}\propto F_{i-1}F_{i-1}^{t}E_{i-1}w_{i}.$$

The score vector $u_{i}$ is not involved directly in the regression model. However, it is important to check if $t_{i}$ and $u_{i}$ support a linear model, for example by a graphical inspection, because the linearity of $t_{i}$ and $u_{i}$ is not in principle guaranteed and must be subsequently evaluated. The plot $t_{i}$ vs. $u_{i}$ could highlight a non-linear behaviour of the responses and, thus, could suggest to transform the responses or to use non-linear methods, such as kernel-PLS2 [14].

### 2.1. Main Properties

The properties of PLS2 derive both from the specific equation used to calculate the weight vectors in 1 and from the properties of the iterative deflation algorithm. They have been discussed by Höskuldsson [15] and are summarized in the following:

- the score vectors of the X-block are a set of mutually orthogonal vectors;
- the weight vectors $w_{i}$ are a set of mutually orthonormal vectors; and
- the matrix $P^{t}W$ is an upper triangular matrix with determinant equal to 1 [11].

In general, the score vectors $u_{i}$ and the columns of the loading matrices P and Q are not sets of mutually orthogonal vectors. Most of the tools developed for model interpretation are based on these properties. For example, the orthogonality of the score vectors has been used to develop the correlation loading plot discussed in Section 3.1, whereas the fact that the weight vectors are mutually orthogonal has driven the formulation of the VIP score introduced in Section 3.3. Moreover, since the matrix $P^{t}W$ is not a diagonal matrix, it can be proved that PLS2 performs an oblique projection of the OLS estimate and the geometry of PLS2 can be investigated under this point of view. For a detailed discussion of this topic the reader can refer to Phatak and de Jong [16].

Another important property that has been discussed in reference 8, which is strictly related to the iterative deflation algorithm, is discussed in the following section.

### 2.2. Structured Noise and Post-Transformation of PLS2 (ptPLS2)

The metabolite content of a biofluid depends on a large number of factors. For example, in a case/control study of a particular disease, the metabolite content of the collected urine samples can depend on age, sex, weight, lifestyle, diet, pathological state, and drug treatment of the recruited subjects. All these factors and the specific factor under investigation (patient/healthy subject) act together to generate data variation. Since PLS2 works by maximizing the covariance in the x-y latent space, the presence of clusters or specific trends in the data, which are not due to the factor of interest, could drive the data projection along directions that are inefficient in explaining the response. Indeed, the covariance between the score vectors $t_{i}$ and $u_{i}$ can be written in terms of correlation and variance as:

$$t_{i}^{t}u_{i}\propto \mathrm{var}{\left(t_{i}\right)}^{1/2}\mathrm{cor}\left(t_{i},u_{i}\right).$$

Thus, the covariance can be maximized through three different strategies: by maximizing the correlation between the vectors $t_{i}$ and $u_{i}$, capturing the linear relationship between X and Y; by maximizing the variance of the score vector $t_{i}$, which provides an explanation of X; by maximizing both covariance and variance. When the covariance is maximized acting on the variance of the scores, PLS2 explains before the variance within the X-block instead of the covariance between predictors and responses. The first latent variables capture the X-block variation while only the next latent variables explain the responses. As a consequence, PLS2 generates an excessive number of latent variables.

The sources of variation that confound PLS2 are called ‘structured noise’ since they can be modelled in terms of latent variables (and then they are ‘structured’ in the data) but behave as a ‘noise’ in that they are unable to explain the response. Two different approaches have been introduced to overcome this unwanted behaviour of PLS2: Orthogonal PLS (OPLS) [10,17] and post-transformation of PLS2 (ptPLS2) [8]. OPLS has been successfully applied to metabolomic problems and, today, is considered as a standard technique. It is implemented in commercial software and freeware packages for data analysis. However, its relationships with PLS2 for multiple Y-response (as in the case of discriminant analysis when more than two classes are considered) have not been investigated in the literature. Here we discuss ptPLS2 that shows clear relationships with PLS2, both for single y- and multiple Y-response, and that is equivalent to OPLS in the case of single y-response. Other methods generating a predictive component equivalent to that of OPLS and ptPLS2 have been proposed for single y-response [18,19].

The original formulation of ptPLS2 is based on the following property of the iterative deflation algorithm: if one uses a non-singular matrix H to transform the weight matrix W as:

$$\tilde{W}=WH,$$

the same residuals of the X- and Y-block and the same matrix B are obtained when $\tilde{W}$ is used within the iterative deflation algorithm instead of W. However, the score structures of the X- and Y-block decomposition can be different from the original ones.

ptPLS2 can be summarised as a three-step procedure: (i) a PLS2 model is built to obtain the weight matrix W; (ii) the weights are subjected to a suitable orthogonal transformation described by the matrix G; and (iii) a new model is built introducing the transformed weights $\tilde{W}=WG$ within the iterative deflation algorithm. If G is calculated according to [8], the new score matrix is composed of two different and independent blocks: one, indicated as $T_{p}$, modelling the data variation correlated to Y and specifying the so called ‘predictive’ or ‘parallel’ part of the model of X, and the other, called $T_{o}$, orthogonal to the Y-block, describing the ‘non-predictive’ or ‘orthogonal’ part of the model. As a result, the following decompositions of the X- and Y-block are obtained:

(4) $$X=T_{p}P_{p}^{t}+T_{o}P_{o}^{t}+E$$

$$Y=T_{p}Q_{p}^{t}+F$$

In the decomposition, $T_{p}$ and $P_{p}$ are the score matrix and the loading matrix of the predictive part of the model of X, respectively, $T_{o}$ and $P_{o}$ the score matrix and the loading matrix of the non-predictive part, and $Q_{p}$ is the loading matrix of the Y-block. The matrix of the regression coefficients is the same as PLS2 and ptPLS2 shows the same goodness-of-fit and power in prediction of PLS2.

ptPLS2 plays a twofold role in metabolomics. Firstly, the response can be explained considering only the predictive part of the model, which usually spans a reduced latent space in comparison with that of the original PLS2 model. Secondly, the investigation of the non-predictive part can highlight sources of variation that are included in the PLS2 model, but that are not related to the response. This helps in the design of new experiments where the factors associated to the non-predictive latent variables are controlled and the factors of interest can be studied with methods based on projection, such as ANOVA-simultaneous component analysis (ASCA) [20] and orthogonally-constrained PLS2 (oCPLS2) [10], without confounding effects.

Recently, a new (but equivalent) formulation based on the scores has been proposed [9].

### 2.3. Orthogonally-Constrained PLS2 (oCPLS2)

Orthogonally-constrained PLS2 (oCPLS2) has been introduced to explicitly include in PLS2 the constraints specified by a well-defined experimental design [10]. Once the main factors acting on the metabolite content of a biological sample are identified and included in an orthogonal design together with the factor of interest, the projection of the residual matrix of the X-block can be driven towards directions orthogonal to the factors that could confound PLS2, focusing the model on the factor under investigation (representing the response). In order to do so, the potential confounding factors are coded in the matrix $Z\in R^{N\times L}$ specifying the *L*-constraints and the scores of the X-block are generated to maximize the covariance with $u_{i}$ being orthogonal to Z. From a mathematical point of view, this requirement can be expressed as in the following maximization problem:

$$w_{i},c_{i}\;s.t.\;\underset{Z^{t}t_{i}={\underline{0}}_{L}}{\arg\max}\;t_{i}^{t}u_{i}=\underset{\begin{array}{l} w_{i}^{t}w_{i}=1\\ c_{i}^{t}c_{i}=1\\ Z^{t}E_{i-1}w_{i}={\underline{0}}_{L} \end{array}}{\arg\max}w_{i}^{t}E_{i-1}^{t}F_{i-1}c_{i}$$

whose solution is obtained solving [10]:

(5) $${\hat{Q}}_{V_{i}}\left(E_{i-1}^{t}F_{i-1}F_{i-1}^{t}E_{i-1}\right)\;{\hat{Q}}_{V_{i}}w_{i}=s_{i}w_{i}$$

The matrix ${\hat{Q}}_{V_{i}}$ is the orthogonal projection matrix that projects any vector v onto the space orthogonal to the column space of $V_{i}$, the latter being the right column orthonormal matrix of the singular value decomposition of $Z^{t}E_{i-1}$.

Substituting Equation (1) with Equation (5) in the ‘eigenvalue’ PLS2 algorithm, the oCPLS2 algorithm is obtained. Since oCPLS2 behaves similarly to PLS2 when structured noise is still present in the data, the post-transformation approach can be applied to improve the identification of the effective latent space.

### 2.4. Non-Linear Problems: Kernel-PLS (KPLS2)

When non-linear relationships between predictors and responses are present in the data, several techniques based on PLS2 have been proposed. Their use depends on the degree of non-linearity. Implicit non-linear latent variable regression (INLR) [21] can be applied in the presence of mild non-linearity whereas in the case of moderate or strong non-linearity PLS based on GIFI-ing (GIFI-PLS) [22] or more complex approaches, such as kernel-PLS (KPLS2) [23] should be applied.

In untargeted metabolomic studies, where the most common scenario is that of a small set of observations (from twenty to one hundred) and a large number of predictors (hundreds or thousands), non-linear modelling is seldom applied because it is difficult to evaluate the presence of over-fitting, and often a subset of the measured predictors shows a non-linear behaviour. In this case, we recommend the use of PLS2, transforming the y-response if needed. However, if the number of predictors is smaller than the number of observations, as in targeted metabolomics investigations, and a robust model validation procedure can be applied, an efficient approach to include non-linearity is to use KPLS2.

In KPLS2, PLS2 regression is not performed directly on the predictors where the problem is non-linear, but it is instead performed on the so called ‘feature space’ obtained transforming the original x-space by suitable functions that turn a non-linear problem into a linear one. Using the formulation of the PLS2 algorithm based on the normalized scores (i.e., the dual formulation of the ‘eigenvalue’ PLS2 algorithm described here) [9], the scalar product in the feature space can be evaluated using kernel functions that make the model easy to calculate. The main disadvantage of KPLS2 is that model interpretation is limited to the investigation of the score space. The evaluation of the role played by the predictors in the model is not straightforward and complex approaches should be applied [24].

The latent space discovered by KPLS2 can be decomposed into predictive and non-predictive subspaces thanks to the application of a suitable post-transformation procedure that works on the scores, called post-transformation of the score matrix (ptLV) [9]. To achieve the same aim, kernel-OPLS (KOPLS) has been introduced [25]. Since the relationships between KOPLS and KPLS2 in the case of multiple Y-response have not been investigated whereas the relationships between KPLS2 and its post-transformed model are known, we suggest the use of KPLS2 and ptLV in the case of datasets with moderate or strong non-linearity.

## 3. Model Interpretation

One of the main advantages of PLS2 with respect to other techniques is the possibility to extract information about the relationships between predictors and responses by direct model interpretation, for example through plots. This is a common opinion between practitioners although it is true only in the presence of mild correlation between the predictors, when the regression coefficients can be directly used for model interpretation. In that case, when the PLS2 model uses a small number of latent variables (two or three latent variables), the w*q plot is an efficient tool, since the relationships between predictors and responses can be investigated using a single plot, also in the case of more than one response [14,26]. On the other hand, in the presence of strong correlation, it is not trivial to obtain a clear and irrefutable interpretation of the model and, in principle, a unique interpretation in terms of the measured predictors may not exist. Indeed, when strong correlations act in the X-block, the regression coefficients do not represent independent effects. Moreover, highly correlated predictors can show different coefficients despite containing the same information. In that case, the regression coefficients cannot be used for model interpretation. Only the latent variables discovered by PLS2 should be considered as they are the only independent predictors able to explain the response. Specifically, the predictive Y-loadings, i.e., the matrix $Q_{p}$, should be investigated to discover the relationships between predictive latent variables and Y-response. However, latent variables may not have a clear physico-chemical meaning, being a linear combination of the measured predictors, while model interpretation in terms of the measured predictors could be requested to support the model from a mechanistic point of view. Consequently, neither regression coefficients nor y-loadings can guarantee the possibility to interpret the model. For this reason, suitable parameters (for example variable influence on projection and selectivity ratio) or procedures (for example stability selection and elimination of uninformative variables in PLS2) have been introduced to allow for model interpretation. The best parameter or procedure to use should be chosen case-by-case because they have been developed considering specific applications and properties of the model. It is important to remark that interpretability is only a way of getting information. Complex models can equally provide accurate and reliable information about the effects of predictors on responses even if they are not interpretable [27].

In the following, we discuss three main questions related to the problem of model interpretation.

### 3.1. Which are the Relationships Between Predictors and Responses?

Two tools are commonly used in metabolomics to discover the relationships between predictors and responses: the w*q plot and the correlation loading plot.

The w*q plot is based on the relationship $B=W^{*}Q^{t}$ that allows the calculation of the regression coefficients from the matrix $W^{*}$ and the loadings of the Y-block. The w* of each predictor (the column of $W^{*}$) and the y-loading q of each response (the columns of Q) are reported in the same plot and, by a suitable geometrical construction, the values of the regression coefficients for a given predictor can be obtained. The use of the w*q plot is described in [14,26] (where it is called w*c plot). It is worth noting that the plot provides reliable information only in the absence of strong multicollinearity and when the model uses only two or three latent variables.

In the correlation loading plot, the Pearson’s correlation coefficients between each (predictive) latent variable and the predictors, and the Pearson’s correlation coefficients between each (predictive) latent variable and the responses are plotted in the same graph. One can use this plot as tool for model interpretation thanks to the orthogonality of the scores. In the plot, the points close to that representing the response of interest, or close to its image obtained by origin reflection, represent the predictors that are positively or negatively correlated to the response. In the presence of strong multicollinearity, we recommend the use of the correlation loading plot, which provides a qualitative explanation of the relationships between predictors and responses (if the goodness-of-fit is high, the relationships become generally quantitative).

### 3.2. How is it Possible to Interpret the Latent Variables in Terms of Single Metabolites?

Given the latent structure of the model, one wants to use single predictors to describe the complexity of the latent structure from a physico-chemical point of view, aiming to obtain a simplified description. The Pearson’s correlation coefficient or the predictive loadings are often used to assess which predictors are the most similar to the predictive latent variables. Recently, Kvalheim and co-workers have introduced a new parameter called Selectivity Ratio (SR) [28,29]. It can be applied only to models with a single predictive latent variable. It is based on the heuristic assumption that the similarity between predictor and latent variable increases with the increasing of the variance explained by the latent variable, and on the possibility to decompose the latent space into two orthogonal subspaces, one predictive and the other orthogonal to the response. Starting from the X-block decomposition 4, SR can be defined for each predictor as the ratio between its variance explained by the predictive latent variable (calculated from $T_{p}P_{p}^{t}$) and its variance unexplained by that latent variable (calculated from $T_{o}P_{o}^{t}+E$). The most interesting variables to be used for explaining the nature of the predictive latent variable are those showing the highest SR. In [29] a method to estimate the threshold to be used for selecting relevant metabolites at a given significance level is described.

### 3.3. Which are the Most Important Metabolites in the Model?

Different strategies have been proposed to evaluate the importance of a predictor in the PLS2 model. In this study we describe only those widely considered in the field to be the most promising. All the presented strategies are based on heuristic definition of importance.

A suitable parameter, called variable influence on projection (VIP score), has been introduced to measure the importance of the variable i in the PLS2 model [30]. VIP is probably the most used parameter to assess the importance of the variable in the PLS2 model. For the x-variable i, the VIP score is defined as:

$${\mathrm{VIP}}_{i}={\left(\frac{P}{\mathrm{SSY}}\sum_{j=1}^{A}W_{ij}^{2}\;{\mathrm{SSY}}_{j}\right)}^{1/2}$$

where ${\mathrm{SSY}}_{j}$ is the sum of squares of Y explained by component j, SSY the total sum of squares of Y explained by the model, A the total number of latent variables, and P the total number of x-variables. It is based on the fact that all the properties of the PLS2 model depend on the weight matrix and that the weight vectors are mutually orthogonal. VIP is largely applied for both model refinement and model interpretation since it provides a ranking for the influence of the x-variables in building the latent space. In the presence of strong correlation, strongly correlated factors show similar VIP while their regression coefficients could be different. In model refinement, variables with VIP less than a certain threshold are excluded. The threshold to be used is usually estimated by cross-validation. It is important to remark that the use of 1 as a threshold should be considered only as a rule of thumb. Indeed, the only justification is the property that 1 is the mean value of the square of VIP.

The VIP score has been adapted to the case of predictive and non-predictive latent variables obtaining the two scores VIP_p_ and VIP_o_, respectively [8]. It is worth noting that variables having high VIPs could show high values of VIP_o_ and low values of VIP_p_. This happens when their main role is to model structured noise instead of explaining the response. Their influence is globally high because their effect is to remove variability that limits PLS2 in finding the right direction to project the data. Moreover, in the presence of structured noise, the exclusion of the variables with the highest VIP could improve the model, because VIP_o_ and VIP could be correlated. In that case, removing variables with high VIP could correspond to removing structured noise.

Instead of calculating specific parameters to assess the importance of the variable, two different procedures have been proposed to select a subset of predictors which are important for the model.

The first one is stability selection. The central idea of stability selection is that real data variations should be present consistently and, therefore, should be found even under perturbation of the data by subsampling or bootstrapping. The procedure has been implemented combining Monte-Carlo sampling and PLS2 with VIP selection [31]. A large number of random subsamples of the data is extracted by Monte-Carlo sampling, and subsequently PLS2 with VIP selection is applied to each subsample. The most important predictors are those selected in more than 50% of the sub-models generated during the stability selection procedure.

The other procedure is called elimination of uninformative variables in the PLS2 model (UVE-PLS) [32]. The idea is to identify the set of uninformative variables by adding artificial noise variables to the collected data. A closed form of the PLS2 model is obtained for the dataset containing the experimental and the artificial variables. The experimental variables that do not have more importance than the artificial variables are eliminated. By doing so, the set of important predictors is selected.

## 4. Applications to Metabolomics

**Figure 2:**
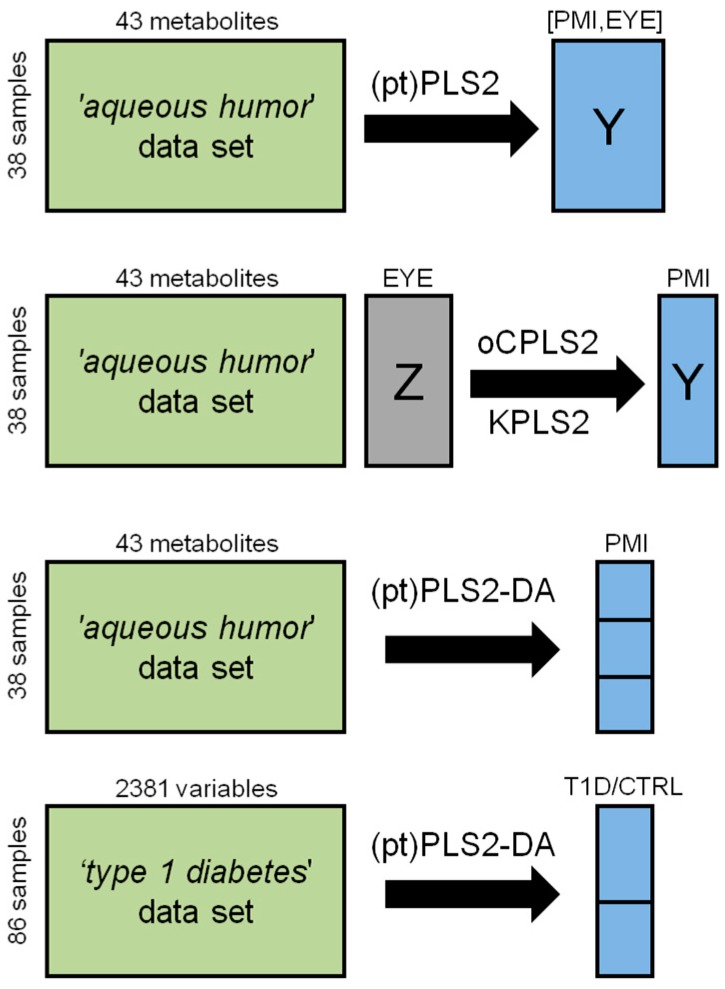
Structure of the data used to present how PLS2-based methods work in practice and techniques applied for data modelling.

In this section, two real datasets are investigated. Figure 2 summarizes the strategies used for modelling the data in the applications discussed in the following. Models and plots have been generated using R-scripts and in-house R-functions implemented by the R 3.3.2 platform (R Foundation for Statistical Computing, Vienna, Austria). In Supplementary Materials the basic R-functions used for model building are reported.

### 4.1. Data Pre-Processing and Data Pre-Treatment

Data pre-processing and data pre-treatment are two steps of the metabolomics workflow that must be applied prior to performing data analysis. Practitioners must specify in detail how the procedures have been applied in order to make the whole workflow reproducible [33]. Accordingly, we recommend the use of a script language (for example based on R programming).

Data pre-processing includes all the procedures that allow the transformation of raw data into a data table. With regards to NMR data, the procedures most commonly used for data pre-processing are: phasing, baseline correction, peak-picking, spectra alignment and binning [34,35]. For GC/U(H)PLC-MS data, data extraction is the tricky step [36].

The main procedures applied in data pre-treatment are briefly described in the following. Missing value imputation is usually required for MS-based metabolomics when data extraction produces undetected peaks or for targeted approaches when the metabolite concentration in some samples is under the limit of quantification. In [37], the approaches employed for missing value imputation are discussed. Data transformation (for example log- or square root transformation) is applied to make the relationships between predictors and responses linear or to remove heteroscedastic noise. Data normalization is used both for correcting dilution differences in biological samples and to reduce the effect of signal loss in long analytical session [38]. Moreover, since PLS2 is sensitive to the scale of the variables, scaling factors are usually applied. In targeted metabolomics, univariate scaling is the most used method whereas Pareto scaling or no scaling are employed in untargeted approaches. Mean centering is usually applied to remove the effect of the center of the data distribution in data projection.

### 4.2. Mulivariate Calibration Problems: PLS2

In multivariate calibration, the relationships between one or more quantitative factors and spectral data (for example UV/IR/NIR data) are investigated. The objective is to find a mathematical model able to predict the factors once the spectral data have been recorded. For its nature, multivariate calibration is an inverse method. Indeed, if from a physico-chemical point of view the factors produce the variation in the spectral data (that are the real responses), in multivariate calibration, one uses spectral data to predict the factors, inverting the role of factor and response. PLS2 is the most used technique to build multivariate calibration models.

Metabolic fingerprints have been successfully used as spectral data to predict toxicity, biological activities, or biological parameters. A metabolic fingerprint is a collection of a very large number of metabolites (from 1000 to 5000) that are (semi-)quantified by high-throughput analytical platforms, such as NMR or GC/U(H)PLC-MS, in a biological sample. An example of application is discussed in the following section.

According to good practice for model building, the optimal number of latent variables to use has been calculated as follows. Different PLS2 models have been generated for an increasing number of latent variables. For each model, Q^2^ (i.e., R^2^ calculated by cross-validation) was calculated applying seven-fold cross-validation and the behavior of the model under permutation tests on the Y-response was investigated (1000 random permutations). We considered the number of latent variables of the model showing the first maximum of Q^2^ under the condition to pass the permutation test as the optimal number of latent variables.

### 4.3. The ‘Aqueous Humor’ Dataset 

Fifty-nine post-mortem aqueous humor (AH) samples were collected from closed and opened sheep eyes at different post-mortem intervals (PMI), ranging from 118 to 1429 minutes. Each sample was analyzed by ^1^H NMR spectroscopy. After raw data processing, Chenomx Profiler (Chenomx, Canada) was applied to obtain a dataset composed of 43 quantified metabolites. A stratified random selection procedure based on the different values of PMI was applied to select the training set (38 samples) and the test set (21 samples). Data were mean centered prior to performing data analysis. More details about sample collection, experimental procedure and data pre-processing can be found in [39].

#### 4.3.1. Design of Experiment and PLS2

**Figure 3:**
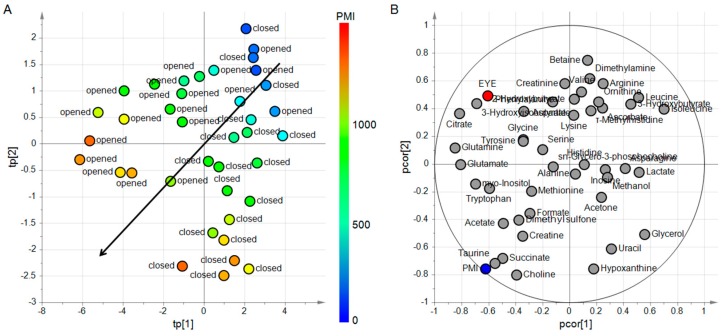
ptPLS2 model built to investigate the effects of PMI and EYE on the metabolite content of AH. In the score scatter plot of panel **A**, each AH sample is represented as a circle with a color code depending on the value of PMI. The arrow indicates the direction where PMI increases. Samples of opened or closed eyes are arranged above or below the arrow, respectively. In the correlation loading plot of panel **B**, the quantified metabolites (light grey circles) and the factors (PMI in blue and EYE in red) are reported in the same plot. It is possible to highlight a group of metabolites (choline, taurine, and succinate) closely related to the effect of PMI whereas other metabolites (citrate, 3-hydroxyisobutyrate, glycerol, and uracil) are related to the effect of EYE.

The experimental design considers two factors: the quantitative factor PMI and the qualitative factor EYE having the two levels opened and closed. Since from each head at a given PMI two samples were collected, one from opened and the other from closed eye, the design resulted an orthogonal design and the effects of PMI and EYE could be investigated without the risk of confounding. To evaluate if these two factors act on the metabolite content of the samples, a PLS2 model was built. We considered the Y-block composed of PMI and EYE whereas the metabolite concentrations were included in the X-block. Considering the training set, the factors were regressed on the set of the 43 metabolites obtaining a PLS2 model with four latent variables, R^2^_PMI_ = 0.96 (*p* = 0.001), R^2^_EYE_ = 0.61 (*p* = 0.001), Q^2^_PMI_ = 0.90 (*p* = 0.001), Q^2^_EYE_ = 0.41 (*p* = 0.001). To better investigate the latent structure of the model, post-transformation was applied to separate the predictive latent subspace described by two predictive latent variables from the non-predictive latent subspace described by two non-predictive latent variables. The score scatter plot and the correlation loading plot of the post-transformed model are reported in Figure 3. The model highlights a strong effect of PMI and a mild effect of EYE on the metabolite content of AH. Moreover, specific subsets of metabolites can be identified as mainly related to the two factors.

If one considers an interaction model including also the term PMI*EYE in the Y-block, the PLS2 analysis highlights a weak effect of also the interaction term (data not shown). Then, if someone wants to predict the PMI given the metabolite content of AH, the effect of the state of eye has to be taken into consideration.

#### 4.3.2. Predicting PMI by oCPLS2

To generate a calibration model for PMI independent of the state of eye, we applied oCPLS2. The matrix Z of the constraints was obtained codifying the qualitative factor opened/closed eye as dummy variable with 0 and 1 depending on the type of sample. PMI was transformed by the square root prior to performing data analysis to take into account a weak non-linearity of the problem. The non-linearity was detected plotting the first latent variable of the model without transformation and the response. After square root transformation, the plot showed a linear behavior between score and response (data not shown). The model showed three latent variables, R^2^_PMI_ = 0.95 (*p* = 0.001), Q^2^_PMI_ = 0.92 (*p* = 0.001), and a standard deviation error in calculation equal to 88 min. The prediction of the test set allowed us to estimate the standard deviation error in prediction that resulted to be equal to 99 min. It is interesting to note that the first latent variable explains only the 26% of the total variance of the response, whereas the second the 68%. This behavior indicates the presence of structured noise. Indeed, only after the projection of the X-block in the subspace orthogonal to the first latent variable, PLS2 is able to find the right direction to obtain efficient projections of the data. Thus, the model was post-transformed to calculate the predictive latent variable that simplified model interpretation.

**Figure 4:**
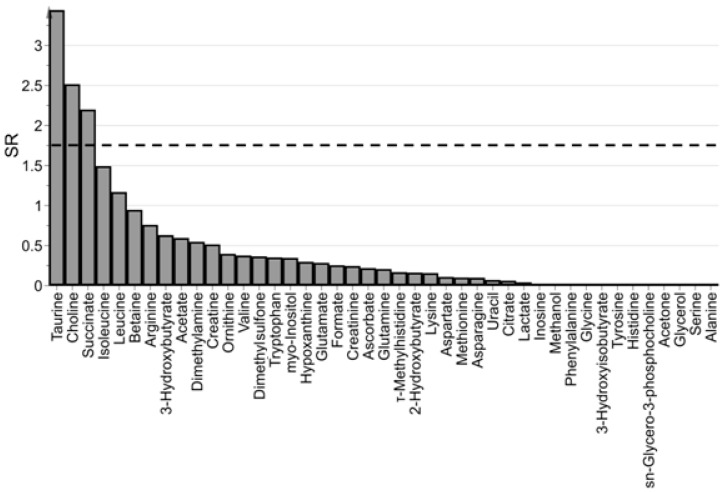
Spectrum of SR; taurine, choline, and succinate resulted significantly related to PMI (α = 0.05); the dashed line indicates the threshold used for variable selection (*F*_(0.05,36,35)_ = 1.75).

Model interpretation was based on the selectivity ratio. In Figure 4 the spectrum of SR is reported. Assuming a significance level α = 0.05 (the threshold for SR resulted *F*_(0.05,36,35)_ = 1.75), three metabolites were selected as mainly related to PMI. It is important to remark that the selected variables should be considered as the most interesting to interpret the predictive latent variable in terms of measured metabolites rather than the single markers of PMI (that should be discovered by univariate methods). As a consequence, the set of selected metabolites can be used as a basis to obtain a simplified explanation of PMI in terms of metabolites. 

Since a weak non-linearity affects the problem, we tried to apply KPLS2 using a second degree polynomial kernel. The KPLS2 model showed 4 latent variables, R^2^_PMI_ = 0.96 (*p* = 0.001), Q^2^_PMI_ = 0.94 (*p* = 0.001), and a standard deviation error in calculation equal to 76 min. The standard deviation error in prediction estimated on the test set resulted to be 98 min. This model performed better than the oCPLS2 model. However, because the test set was too small to allow a robust model validation for a kernel method, and KPLS2 does not provide a mechanistic view of the process under investigation, we did not consider the improvements in performance so relevant to justify the use of a kernel method.

### 4.4. Classification Problems: PLS2-DA

PLS2 has been developed to perform linear regression. However, the following two-step procedure can be applied to obtain a classifier based on PLS2. In the first step, a PLS2 model is built regressing a suitable dummy Y-response specifying the class membership on the predictors whereas, in the second step, the latent variables calculated by PLS2 (or by ptPLS2) are used to build a classical classifier, for example a naive Bayes classifier, or are used as variables for linear discriminant analysis (LDA). The model obtained in the first step is called PLS2-DA (or simply PLS-DA). It is worth noting that without the second step, it is not possible to establish the class membership of an observation because it is necessary to define rules or procedures to interpret the predictions of PLS2-DA in terms of class. As a consequence, when one uses PLS2-DA, it is necessary to specify the rules used for assessing the class membership. [40] is a well-written tutorial on this topic.

#### 4.4.1. Dummy Y-Response and Scaling

The formulation of PLS2-DA is based on a dummy Y-response composed of ones and zeros used to specify the class membership. For a G-class problem, the Y-matrix is built considering G columns, being each column associated to a particular class. An observation belonging to class *i* is codified with 1 in column *i* and zero in the other columns. Thus, the Y-matrix is autoscaled (i.e., univariate scaled and mean centered) before the application of PLS2. This formulation is heuristic. It has been justified by the analogy with LDA, which can be obtained regressing the dummy Y-response on the X-matrix by OLS.

In the case of balanced classes, PLS2-DA calculates latent variables that maximize the among-groups variation. However, in the general case of unbalanced classes, the maximization problem 3 becomes a complex function of the number of samples of each class and the among-groups variation is not maximized, except for a two-class problem where the among-groups variation is still maximized.

Barker and Rayens investigated the mathematical framework of PLS2-DA and suggested to modify the scaling in order to obtain a more formal statistical explanation [41].

#### 4.4.2. Application to the ‘Aqueous Humor’ Dataset

Considering the training set of the ‘aqueous humor’ dataset, the range of PMI was split into three intervals corresponding to PMI less than 500 min (10 samples), PMI from 500 to 1000 min (14 samples) and PMI greater than 1000 min (14 samples). The objective was to investigate the changes in the metabolite content of AH during these three intervals. Since R^2^ and Q^2^ are not suitable parameters in classification because the response is a qualitative variable, we consider the Cohen’s kappa. Specifically, in the model parameter optimization step, we considered the Cohen’s kappa in calculation (k) and the Cohen’s kappa calculated by seven-fold cross-validation (k_7-fold_). The number of latent variables of the PLS2-DA model was determined on the basis of the first maximum of k_7-fold_ under the condition to pass the permutation test on the Y-response (1000 random permutations). The class membership was assessed applying LDA to the scores of the PLS2-DA model.

The PLS2-DA model showed three latent variables and the classifier k = 0.96 (*p* = 0.001) and k_7-fold_ = 0.96 (*p* = 0.001). The test set was predicted misclassifying three samples (k in prediction = 0.77).

**Figure 5:**
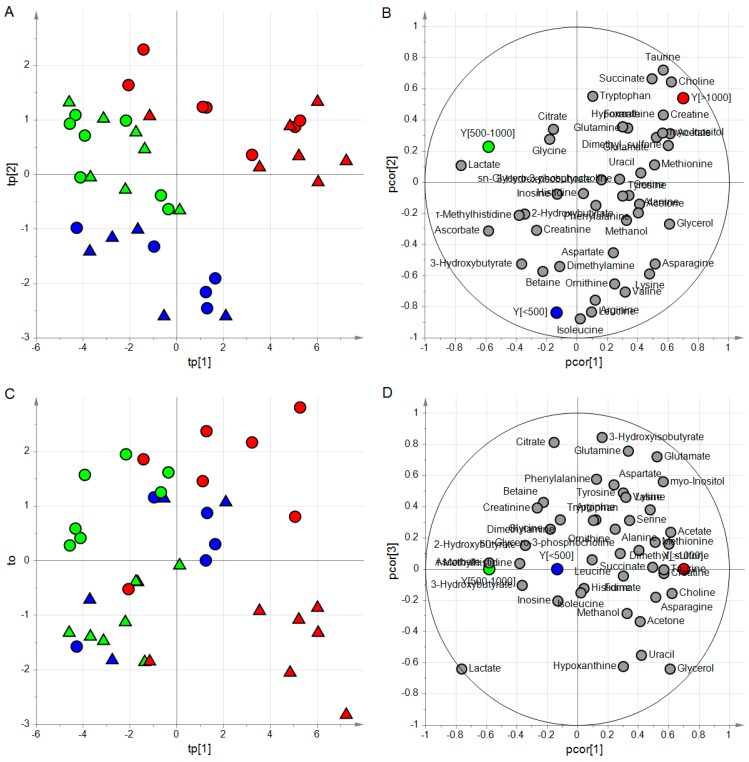
ptPLS2-DA model; the two predictive latent variables tp[1] and tp[2] are reported in the score scatter plot of panel **A** where the samples of the three classes result to belong to three different regions; in the correlation loading plot of panel **B**, groups of metabolites characterizing each class can be identified; if one considers the orthogonal latent variable to, samples of opened eyes show positive values while samples of closed eyes negative values (panel **C**); the correlation loading plot of panel **D** allows the identification of metabolites related to the state of the eye; samples with PMI < 500 min are colored in blue, samples with PMI between 500 and 1000 min in green and samples with PMI > 1000 min in red; triangles are used for samples of closed eyes whereas circles for opened eyes.

The investigation of the score scatter plots and the correlation loading plots of the PLS2-DA part of the classifier after post-transformation (Figure 5) provided a simplified characterization of the three classes in terms of metabolites. Specifically, isoleucine and leucine presented the highest levels for PMI < 500 min, lactate resulted to be higher for PMI in the range [500,1000] min than in the other two classes, and choline, creatine, acetate, dimethylsulfone, succinate, and taurine showed the highest levels for PMI > 1000 min. The orthogonal latent variable could be interpreted as representing the effects of the state of the eye, and resulted to be mainly correlated to 3-hydroxyisobutyrate, citrate, glutamine, and glycerol.

#### 4.4.3. Application to the ‘Type 1 Diabetes’ Dataset

The dataset was generated analyzing the urine samples of 56 patients with type 1 diabetes and 30 healthy controls by UPLC-MS. A clinical examination, including pubertal stage evaluation according to Tanner scale, was conducted and a detailed family and personal medical history was collected for all the enrolled subjects.

For the accurate detection of metabolites in the samples, the compounds separated in the UPLC were ionized and analyzed through a quadrupole time-of-flight (QToF) mass spectrometer. The electrospray ionization source operated either in positive and negative mode. Raw data were extracted by MarkerLynx software (Waters, Milford, MA, USA). Probabilistic quotient normalization, log-transformation, and mean centering were applied prior to performing data analysis. Two datasets, one corresponding to the positive ionization mode with 2381 variables (POS dataset) and the other to the negative ionization mode with 1435 variables (NEG dataset), were obtained. More details about sample collection, experimental procedure and data pre-processing can be found in [42]. The aim of the study was to compare the urinary metabolome of the two groups of subjects. Specifically, the POS dataset has been investigated as an example of short and wide dataset.

Since urinary metabolome could be affected by weight, sex, age and pubertal stage, we investigated whether the two groups showed differences in these parameters. Assuming a significance level α = 0.05, we did not find differences between the two groups.

Stability selection based on Monte-Carlo sampling and PLS2-DA with VIP selection has been applied to select a subset of relevant metabolites useful to discriminate the two groups [31]. One-hundred random subsamples of the collected samples were extracted by Monte-Carlo sampling (with a prior probability of 0.70), and then PLS2-DA with VIP selection was applied to each subsample, obtaining a set of 100 discriminant models. For each model, the number of latent variables and the threshold to use for VIP selection were optimized on the basis of the maximum value of k_7-fold_. The class membership was determined by LDA applied on the scores of the PLS2-DA model. The predictors selected in more than 50 models were considered as relevant. Moreover, the performance in prediction of each model was estimated predicting the outcomes of the samples excluded during subsampling.

**Figure 6:**
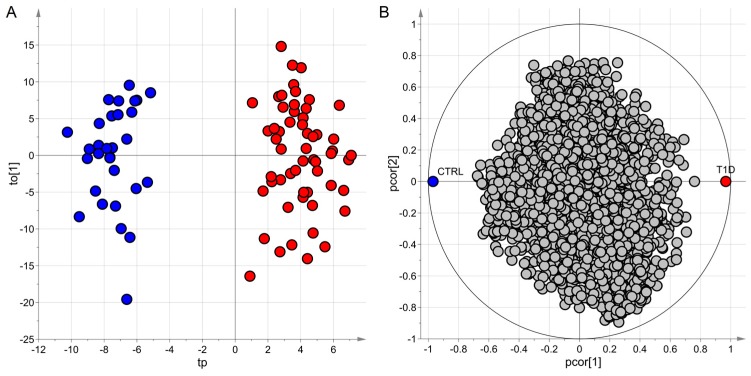
ptPLS2-DA model; the predictive latent variables tp and the first non-predictive latent variable to[1] are reported in the score scatter plot of panel **A** where the samples of the two classes belong to two different regions; in the correlation loading plot of panel **B**, groups of metabolites characterizing each class can be identified; samples of subjects with type 1 diabetes (T1D) are colored in red whereas samples of the control group (CTRL) in blue.

A total of 318 variables were selected as relevant, and 279 showed q for the t-test (we have applied a false discovery rate according to the Storey method) less than 0.20 and area under the receiver operating characteristic curve greater than 0.50 (significance level α = 0.05). After variable annotation, metabolites mainly represented by gluco- and mineral-corticoids, phenylalanine and tryptophan catabolites, small peptides, and gut bacterial products showed higher level in children with type 1 diabetes. The Cohen’s kappa in prediction resulted 0.94 (95% CI = 0.78–1.00). In Figure 6 the score scatter plot and the correlation loading plot of the ptPLS2-DA model obtained considering the whole dataset are reported. The model showed one predictive latent variable and two non-predictive latent variables, and the classifiers k = 1.00 (*p* = 0.001) and k_7-fold_ = 0.92 (*p* = 0.001). The predictive latent variable did not show significant correlation (significance level α = 0.05) with weight, sex, age, and Tanner index.

## 5. Conclusions

PLS2 is a heuristic regression method that combines the measured predictors into suitable latent variables to perform least squares regression in the latent space. It solves many of the problems related to the structure of the metabolomic data. Indeed, it is robust in the presence of multicollinearity, redundancy, and noise, and can be applied to short and wide matrices.

Since PLS2 could include unnecessary sources of variation in the model, the possibility to distinguish predictive and non-predictive latent variables simplifies model interpretation and allows a better estimation of the effects in the regression model.

When a well-defined experimental design is implemented, orthogonal constraints can be included in the maximization problem of PLS2 in order to remove specific sources of variation that could confound data projection. In this way, general models independent of specified factors can be generated.

However, further investigations are needed to deal with some important issues.

Since in PLS2 the exact estimation of the prediction error is hampered by the non-linearity of the regression coefficients with respect to the response and a model in the statistical sense is not formulated, it is difficult to put PLS2 in the general framework of linear regression modelling. Then, it is necessary to investigate how to bridge the gap with traditional methods and clarify why the maximization problem solved by PLS2 works so well in practice.

Moreover, new parameters or procedures for model interpretation should be developed to better clarify the role played by the metabolites in the model when strong multicollinearity affects the data.

Finally, the concept of applicability domain should be introduced and adapted to the needs of metabolomics. This is fundamental to state whether the assumptions of the model are met and for which new samples the model can be reliably applied, avoiding extrapolation.

## Supplementary Materials

The following are available online at https://www.mdpi.com/2218-1989/9/3/51/s1.
