# Supplementary material for: PLS2 in Metabolomics
Source: Metabolites. 2019 Mar 15;9(3):51. doi: 10.3390/metabo9030051 (PMC6468483; doi:10.3390/metabo9030051)
Supplement: Supplementary file 1 [file metabolites-09-00051-s001.pdf]

Supplementary Materials:

PLS2 IN METABOLOMICS

Matteo Stocchero, Emanuela Locci, Ernesto d'Aloja, Matteo Nioi, Eugenio Baraldi and Giuseppe Giordano

Basic R-functions

PLS2 and ptPLS2

Input: matrix X of the predictors, matrix Y of the responses, number A of latent variables of the model.

Output: score matrix T, score matrix of the post-transformed model Tpt, weight matrix W, matrix Wstar (W*), matrix of the regression coefficient B.

```
PLS2<-function(X,Y,A) {
        W<-matrix(rep(0,A*ncol(X)),nrow=ncol(X),ncol=A)
        P<-matrix(rep(0,A*ncol(X)),nrow=ncol(X),ncol=A)
        T<-matrix(rep(0,A*nrow(X)),nrow=nrow(X),ncol=A)
        E<-X
        for (i in 1:A) {
                W[,i]<-svd(t(Y)%*%E)$v[,1]
                T[,i]<-E%*%W[,i]
                P[,i]<-t(E)%*%T[,i]/sum(T[,i]*T[,i])
                E<-E-T[,i]%*%t(P[,i])
        }
        Ws<-W%*%solve(t(P)%*%W)
        B<-Ws%*%solve(t(T)%*%T)%*%t(T)%*%Y
        d1<-svd(t(Y)%*%X%*%W)
        Np<-length(which(d1$d>10^-8))
        G<-step.2(X,Y,W)
        if (A<=Np) Tpt<-T
        if (A>Np) Tpt<-step.3(X,Y,W,G)$Tpt
        pls2<-list(T=T,Tpt=Tpt,W=W,Wstar=Ws ,B=B)
        return(pls2)
}
step.2<-function(X,Y,W) {
        G<-matrix(rep(0,ncol(W)*ncol(W)),ncol=ncol(W))
        I<-diag(rep(1,ncol(W)))
        d1<-svd(t(Y)%*%X%*%W)
        N<-length(which(d1$d>10^-8))
        V<-Re(d1$v[,1:N])
```

```
        d2<-eigen((I-V%*%t(V))%*%(t(W)%*%t(X)%*%X%*%W))
        M<-length(which(Re(d2$values)>10^-8))
        Go<-Re(d2$vectors[,1:M])
        Gp<-Re(eigen(t(I-Go%*%t(Go))%*%(I-Go%*%t(Go))) $vectors[,1:(ncol(W)-M)])
        G<-cbind(Go,Gp)
        return(G)
}
step.3<-function(X,Y,W,G) {
        Tpt<-matrix(rep(0,nrow(X)*ncol(W)),ncol=ncol(W))
        E<-X
        F<-Y
        Wpt<-W%*%G
        for (i in 1:ncol(W)) {
                Tpt[,i]<-E%*%Wpt[,i]
                Q<-Tpt[,i]%*%t(Tpt[,i])/sum(Tpt[,i]^2)
                E<-E-Q%*%E
                F<-F-Q%*%F
        }
        Ppt<-t(X)%*%Tpt%*%solve(t(Tpt)%*%Tpt)
        Wspt<-Wpt%*%solve(t(Ppt)%*%Wpt)
        ptmodel<-list(Tpt=Tpt,Wpt=Wpt,Wspt=Wspt)
        return(ptmodel)
}
```

oCPLS2

Input: matrix X of the predictors, matrix Y of the responses, matrix Z of the constraints, number A of latent variables of the model.

Output: score matrix T, score matrix of the post-transformed model Tpt, weight matrix W, matrix Wstar (W*), matrix of the regression coefficient B.

```
oCPLS2<-function(X,Y,Z,A) {
        W<-matrix(rep(0,A*ncol(X)),nrow=ncol(X),ncol=A)
        P<-matrix(rep(0,A*ncol(X)),nrow=ncol(X),ncol=A)
        T<-matrix(rep(0,A*nrow(X)),nrow=nrow(X),ncol=A)
        B<-t(Z)%*%X
        h<-svd(B)
        R<-length(which(h$d>10^-8))
        V<-h$v[,1:R]
        Q<-diag(rep(1,ncol(X)))-V%*%t(V)
        E<-X
        F<-Y
        for (i in 1:A) {
```

```
        W[,i]<- svd(t(F)%*%E%*%Q)$v[,1]
        T[,i]<-E%*%W[,i]
        P[,i]<-t(E)%*%T[,i]/sum(T[,i]*T[,i])
        E<-E-T[,i]%*%t(P[,i])
        F<-F-T[,i]%*%t(T[,i])%*%F/sum(T[,i]*T[,i])
    }
    Ws<-W%*%solve(t(P)%*%W)
    B<-Ws%*%solve(t(T)%*%T)%*%t(T)%*%Y
    d1<-svd(t(Y)%*%X%*%W)
    Np<-length(which(d1$d>10^-8))
    G<-step.2(X,Y,W)
    if (A<=Np) Tpt<-T
    if (A>Np) Tpt<-step.3(X,Y,W,G)$Tpt
    ocpls2<-list(T=T,Tpt=Tpt,W=W,Wstar=Ws,B=B)
    return(ocpls2)
}
```

KPLS2

Input: matrix X of the predictors, matrix Y of the responses, number A of latent variables of the model, (a, b, c) parameters of the polynomial kernel $k(x,y)=(a(x^{t}y)+b)^{p}$, matrix Xtest of the test set to be predicted

Output: normalized score matrix Tn, normalized score matrix of the test set Tnpred, calculated response Ycalc, predicted response Ypred

```
KPLS2<-function(X,Y,A,a,b,p,Xtest) {
    Tn<-matrix(rep(0,nrow(X)*A),ncol=A)
    U<-matrix(rep(0,nrow(Y)*A),ncol=A)
    K<-K.matrix(X,a,b,p)
    F<-Y
    for (i in 1:A) {
        Tn[,i]<-Re(eigen(K%*%F%*%t(F))$vectors[,1])
        U[,i]<-F%*%t(F)%*%Tn[,i]
        Q<-diag(1,nrow(X))-Tn[,i]%*%t(Tn[,i])
        K<-Q%*%K%*%Q
        F<-Q%*%F
    }
    Ymod<-Y-F
    K<-K.matrix(X,a,b,p)
    Kp<-K.matrixpred(Xtest,X,a,b,p)
    Ypred<-Kp%*%U%*%solve(t(Tn)%*%K%*%U)%*%t(Tn)%*%Y
    Tnpred<-Kp%*%U%*%solve(t(Tn)%*%K%*%U)
    r<-list(Tn=Tn ,Tnpred=Tnpred, Ycalc=Ymod,Ypred=Ypred)
    return(r)
```

```
}
K.matrix<-function(X,a,b,p) {
    Km<-matrix(rep(-999,nrow(X)*nrow(X)),nrow=nrow(X))
    for (i in 1:nrow(X)) {
        for (j in 1:nrow(X)) {
            Km[i,j]<-(a*t(X[i,])%*%X[j,]+b)^p
        }
    }
    p<-matrix(rep(1,nrow(X)),ncol=1)
    Qc<-diag(1,nrow(X))-(1/nrow(X))*p%*%t(p)
    Km<-Qc%*%Km%*%Qc
    return(Km)
}
K.matrixpred<-function(X1,X2,a,b,p) {
    Kp<-matrix(rep(-999,nrow(X1)*nrow(X2)),nrow=nrow(X1))
    K<-matrix(rep(-999,nrow(X2)*nrow(X2)),nrow=nrow(X2))
    for (i in 1:nrow(X1)) {
        for (j in 1:nrow(X2)) {
            Kp[i,j]<-(a*t(X1[i,])%*%X2[j,]+b)^p
        }
    }
    for (i in 1:nrow(X2)) {
        for (j in 1:nrow(X2)) {
            K[i,j]<-(a*t(X2[i,])%*%X2[j,]+b)^p
        }
    }
    p<-matrix(rep(1,nrow(X2)),ncol=1)
    pp<-matrix(rep(1,nrow(X1)),ncol=1)
    Qc<-diag(1,nrow(X2))-(1/nrow(X2))*p%*%t(p)
    P<-(1/nrow(X2))*pp%*%t(p)
    Kp<-(Kp-P%*%K)%*%Qc
    return(Kp)
}
```
